# Prevalence, Morbidity, and Mortality of Men With Sex Chromosome Aneuploidy in the Million Veteran Program Cohort

**DOI:** 10.1001/jamanetworkopen.2024.4113

**Published:** 2024-03-29

**Authors:** Shanlee M. Davis, Craig Teerlink, Julie A. Lynch, Bryan R. Gorman, Meghana Pagadala, Aoxing Liu, Matthew S. Panizzon, Victoria C. Merritt, Giulio Genovese, Judith L. Ross, Richard L. Hauger

**Affiliations:** 1 Department of Pediatrics, University of Colorado School of Medicine, Aurora; 2 eXtraOrdinarY Kids Clinic, Children’s Hospital Colorado, Aurora; 3 VA Informatics and Computing Infrastructure, VA Salt Lake City Healthcare System, Salt Lake City, Utah; 4 Department of Internal Medicine, University of Utah Health, Salt Lake City; 5 College of Nursing and Health Sciences, University of Massachusetts, Lowell; 6 VA Boston Healthcare System, Boston, Massachusetts; 7 Medical Scientist Training Program, University of California San Diego, La Jolla; 8 Biomedical Science Program, University of California San Diego, La Jolla; 9 University of Helsinki Institute for Molecular Medicine, Helsinki, Finland; 10 Center for Behavioral Genetics of Aging, School of Medicine, University of California San Diego, La Jolla; 11 Department of Psychiatry, University of California San Diego, La Jolla; 12 Center of Excellence for Stress and Mental Health, VA San Diego Healthcare System, San Diego, California; 13 Program in Medical and Population Genetics, Broad Institute of MIT and Harvard, Boston, Massachusetts; 14 Stanley Center for Psychiatric Research, Broad Institute of MIT and Harvard, Cambridge, Massachusetts; 15 Department of Genetics, Harvard Medical School, Boston, Massachusetts; 16 Nemours Children’s Hospital Delaware, Wilmington; 17 Department of Pediatrics, School of Medicine, Thomas Jefferson University, Philadelphia, Pennsylvania

## Abstract

**Importance:**

The reported phenotypes of men with 47,XXY and 47,XYY syndromes include tall stature, multisystem comorbidities, and poor health-related quality of life (HRQOL). However, knowledge about these sex chromosome aneuploidy (SCA) conditions has been derived from studies in the less than 15% of patients who are clinically diagnosed and also lack diversity in age and genetic ancestry.

**Objectives:**

To determine the prevalence of clinically diagnosed and undiagnosed X or Y chromosome aneuploidy among men enrolled in the Million Veteran Program (MVP); to describe military service metrics of men with SCAs; and to compare morbidity and mortality outcomes between men with SCA with and without a clinical diagnosis vs matched controls.

**Design, Setting, and Participants:**

This cross-sectional study used a case-control recruitment design to select biological males enrolled in the MVP biobank in the US Veterans Administration health care system from 2011 to 2022. Cases were participants with 47,XXY syndrome or 47,XYY syndrome, matched 1:5 with controls based on sex, age, and genetic ancestry. Data were analyzed from January 2022 to December 2023.

**Exposure:**

Genomic identification of an additional X or Y chromosome

**Main Outcomes and Measures:**

Outcomes of interest included prevalence of men with SCAs from genomic analysis; clinical SCA diagnosis; Charlson Comorbidity Index; rates of outpatient, inpatient, and emergency encounters per year; self-reported health outcomes; and standardized mortality ratio.

**Results:**

Of 595 612 genotyped males in the MVP, 862 had an additional X chromosome (47,XXY) and 747 had an extra Y chromosome (47,XYY), with the highest prevalence among men with East Asian (47,XXY: 10 of 7313 participants; 47,XYY: 14 of 7313 participants) and European (47,XXY: 725 of 427 143 participants; 47,XYY: 625 of 427 143 participants) ancestry. Mean (SD) age at assessment was 61 (12) years, at which point 636 veterans (74.X%) with 47,XXY and 745 veterans (99%) with 47,XYY remained undiagnosed. Individuals with 47,XXY and 47,XYY had similar military service history, all-cause standardized mortality ratio, and age of death compared with matched controls. Individuals with SCA, compared with controls, had higher Charlson Comorbidity Index scores (47,XXY: mean [SD], 4.30 [2.72] vs controls: mean [SD], 3.90 [2.47]; 47,XYY: mean [SD], 4.45 [2.90] vs controls: mean [SD], 3.82 [2.50]) and health care utilization (eg, median [IQR] outpatient encounters per year: 47,XXY, 22.6 [11.8-37.8] vs controls, 16.8 [9.4-28]; 47,XYY: 21.4 [12.4-33.8] vs controls: 17.0 [9.4-28.2]), while several measures of HRQOL were lower (eg, mean [SD] self-reported physical function: 47,XXY: 34.2 [12] vs control mean [SD] 37.8 [12.8]; 47,XYY: 36.3 [11.6] vs control 37.9 [12.8]). Men with a clinical diagnosis of 47,XXY, compared with individuals without a clinical diagnosis, had higher health care utilization (eg, median [IQR] encounters per year: 26.6 [14.9-43.2] vs 22.2 [11.3-36.0]) but lower Charlson Comorbidity Index scores (mean [SD]: 3.7 [2.7] vs 4.5 [4.1]).

**Conclusion and Relevance:**

In this case-control study of men with 47,XXY and 47,XYY syndromes, prevalence of SCA was comparable with estimates in the general population. While these men had successfully served in the military, they had higher morbidity and reported poorer HRQOL with aging. Longer longitudinal follow-up of this sample will be informative for clinical and patient-reported outcomes, the role of ancestry, and mortality statistics.

## Key Points

**Question:**

What is the prevalence of an additional X or Y chromosome among men who served in the US military, and what morbidity outcomes are associated with these conditions?

**Findings:**

In this cross-sectional study of 595 612 men in the Million Veteran Program, 1 in 370 men had a sex chromosome aneuploidy, but only 14.2% were clinically diagnosed by age 60 years. Medical morbidity and health care utilization metrics were higher for men with either 47,XXY or 47,XYY compared with matched controls.

**Meaning:**

These findings suggest that men with sex chromosome aneuploidies successfully serve in the US military but experienced profound underdiagnosis and higher medical morbidity with aging.

## Teaser

This cross-sectional study assesses the prevalence of morbidity and mortality associated with 47,XXY and 47,XYY karyotypes compared with matched controls among participants in the Million Veteran Program.

## Introduction

Approximately 1 in 400 males have an extra X or Y chromosome, resulting in approximately 5000 males born in the US annually with 47,XXY (Klinefelter syndrome) or 47,XYY (double-Y syndrome).^1^ These male sex chromosome aneuploidy (SCA) conditions are associated with broad multisystem medical and neuropsychiatric comorbidities; however, due to substantial phenotypic variability and lack of pathognomonic dysmorphic features, less than 25% of men with 47,XXY and less than 10% of men with 47,XYY receive a diagnosis.^1^ With few exceptions, SCA research has been limited to the minority of individuals with clinically ascertained diagnosis, leading to substantial bias and limited knowledge of the full spectrum of health and quality of life (QOL) outcomes for individuals with 47,XXY or 47,XYY. In addition, much SCA research originates from Western European countries, which lack the racial, ethnic, and socioeconomic diversity present in the US.

Population registries of clinically ascertained men with SCAs in European countries report a decreased lifespan and higher incidence rates for nearly all clinical diagnostic codes compared with sex- and age-matched controls.^2,3,4,5,6^ A 2022 study using the UK Biobank identified 356 men via genotype array to have X or Y chromosome polysomy.^7^ Most of the findings in the study by Zhao et al^7^ aligned with prior data, including an 85% nondiagnosis rate and worse overall health outcomes compared with men with 46,XY from both self-report and medical record data sources. In addition to increased morbidity and mortality outcomes, a meta-analysis summarizing the published literature on QOL in men with 47,XXY found a negative association with physical, psychological, and social QOL domains.^8^ However, on the individual level, the phenotypic result of an additional sex chromosome is variable, and the net impact of a clinical diagnosis remains uncertain.

We used the Veteran’s Health Administration (VHA) Million Veteran Program (MVP), a voluntary population-based study of genetic determinants of various illnesses and health outcomes for individuals who have served in the US military,^9^ to address limitations in existing 47,XXY and 47,XYY research. The aims of our investigation were to determine the prevalence of male participants in MVP with X or Y chromosome aneuploidy overall, both with and without a clinical diagnosis; compare military service and health outcomes for participants with SCAs with matched controls; and compare outcomes between participants with and without a clinical diagnosis.

## Methods

The MVP study was approved by the VHA central institutional review board, and all individuals provided written informed consent prior to study enrollment. This study adheres to the Strengthening the Reporting of Observational Studies in Epidemiology (STROBE) reporting guideline.

### Overview of Study Design

**Figure 1.**
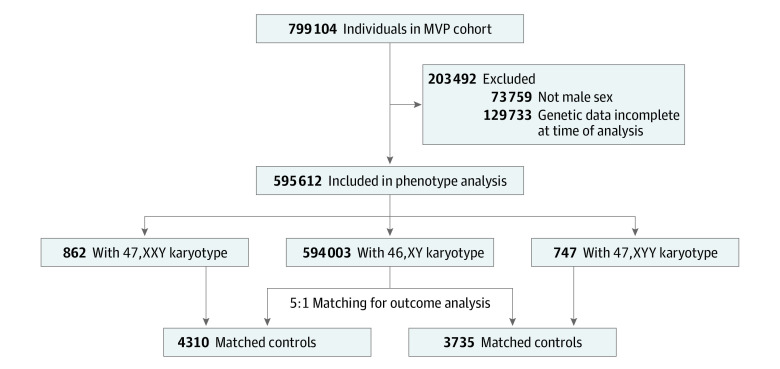
Cohort Selection Flowchart Flow diagram of Million Veteran Program (MVP) participants included in the current analysis.

This cross-sectional, case-control study uses data from the MVP (which has been described elsewhere^9^) and analyzed genotype, VHA electronic health records (EHRs), and survey responses of 658 582 men (Figure 1). Veterans enrolled in MVP provide a blood sample for genotyping, grant access to their EHR for research, and complete the baseline and lifestyle surveys.^10^ Survey response rates in our cohort were similar to MVP metrics as a whole.^11^

### Genotyping, Ancestry, and SCA Determination

Blood samples are banked at the VHA Central Biorepository at the Massachusetts Veterans Epidemiology Research and Information Center. DNA is extracted from peripheral blood leukocytes and analyzed on the MVP custom Axiom Biobank 1.0 array (Affymetrix) consisting of 723 305 unique SNPs, as described previously.^12^ Genotype data within the GenISIS workspace were used to determine genetic ancestry using the harmonized ancestry and race ethnicity (HARE) method, which combines genetic ancestry data with self-identified race and ethnicity, classified as African, East Asian, European, Hispanic, and other (eg, American Indian or Alaska Native, Native Hawaiian or Other Pacific Islander, and multiples races or ethnicities) or unavailable.^13^ To identify men with an SCA, the sex chromosome dosage was estimated by array intensity measurements. Normalized probeset intensities (log-R ratios [LRRs]) were calculated with 20 170 nonpseudoautosomal X probesets and 157 non-pseudoautosomal Y probesets. Comparison of median LRRs on X and Y identified clear clusters corresponding to samples with 46,XY, 47,XXY and 47,XYY karyotypes. A final SCA call set was then obtained by thresholding median LRR for X and median LRR for Y around each identified cluster (Figure 1B).

### Defining the Analytic Cohort

Male individuals with available VA EHRs who were within the 47,XXY or 47,XYY clusters defined our cases for this analysis, and individuals in the 46,XY cluster were controls. Analyses were not stratified by race or ethnicity because presence of an additional sex chromosome was considered as a single genetic variant eliminating the possibility of population stratification due to genetic drift influencing statistical tests. Therefore, we proceeded with a matched analysis in which each male with an extra X or Y chromosome was randomly matched with five 46,XY males on age at time of enrollment and genetic ancestry based on HARE designation.^14^

### Outcomes

We determined the prevalence of SCA based on the number of males with a second X or Y chromosome over the total number of males in MVP. To determine whether an individual with a genetically identified 47,XXY or 47,XYY karyotype had a clinical diagnosis of SCA, we queried *International Classification of Diseases, Ninth Revision *(*ICD-9*) and *International Statistical Classification of Diseases and Related Health Problems, Tenth Revision *(*ICD-10*) diagnosis codes consistent with SCA (*ICD-9*: 9 758.7-81; *ICD-10*: Q98.0-9). Participants with at least 1 diagnostic code were determined to be clinically diagnosed, and age at the first diagnosis code was retrieved.

Descriptive military service metrics were obtained from a combination of Department of Veteran Affairs records and survey responses. We calculated health care utilization metrics, including the number of outpatient, emergency, and overnight hospital encounters per year, with duration of follow-up determined as the difference between the initial and most recent encounter dates. The primary measure of medical morbidity was the Charlson Comorbidity Index (CCI), a calculated metric incorporating multiple medical conditions obtained from the EHR validated to estimate 10-year survival.^15^ Additional measures of morbidity included self-reported number of prescription medications, self-reported past medical history, and validated summary scores from the baseline and lifestyle surveys, as previously described,^10^ including the Veterans RAND 12-Item Short-Form Health Survey^16,17^ as a measure of physical and mental health-related QOL, Medical Outcomes Study Cognitive Functioning-Revised Scale^18,19^ as a subjective estimate of cognitive difficulties, and the Patient Health Questionnaire-4^20^ and the Posttraumatic Stress Disorder Checklist^21^ as estimates of current psychological functioning.

Finally, we calculated the standardized mortality ratio (SMR) as the number of deaths in cases divided by the expected number of deaths based on matched controls within the same time period. The primary cause of death was recoded into 1 of 39 categories according to the National Center for Health Statistics manual.^22^

### Statistical Analysis

For the first aim, prevalence of 47,XXY and 47,XYY per 100 000 males was calculated; the proportion of individuals with a clinical diagnosis was also calculated. For the second aim, we conducted outcome comparisons between cases (individuals with 47,XXY or 47,XYY) vs their matched controls using *t* tests or Wilcoxon signed-rank tests, as appropriate. We used χ^2^ tests (or Fisher exact if cell size <10) for categorical variables, and calculated odds ratios (ORs) with 95% CIs. We used the same approach to compare individuals with and without a clinical SCA diagnosis. To avoid type 1 error with a large sample size and multiple comparisons, we elected a conservative 2-sided α = .005 for primary outcomes. We kept a 2-sided α = .05 for exploratory, hypothesis-generating outcomes to avoid type 2 error. Analyses were conducted using R statistical software version 4.0.3 (R Project for Statistical Computing), and figures were created with GraphPad version 9.5.1 (Prism). Data were analyzed from January 2022 to December 2023.

## Results

**Figure 2.**
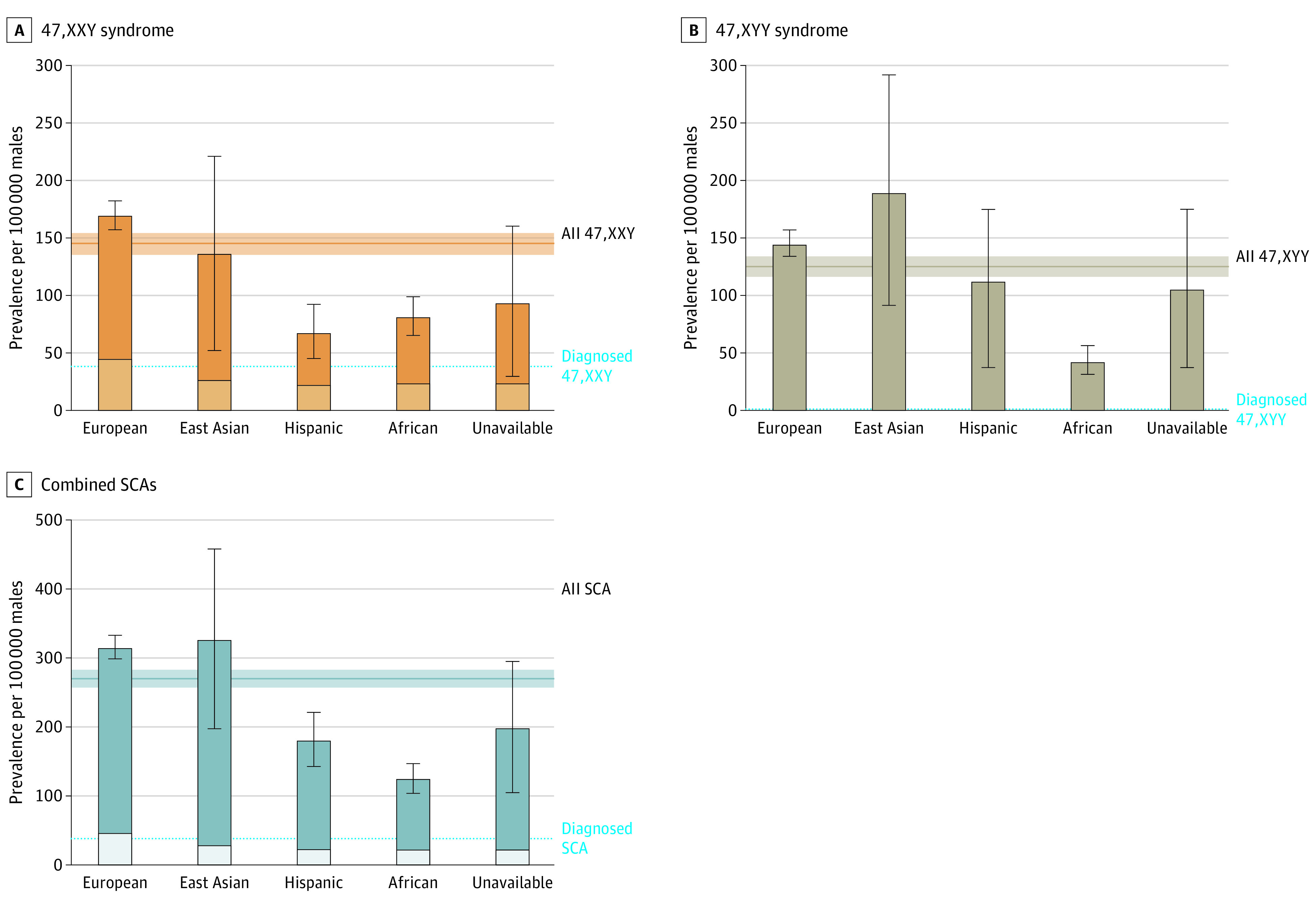
Prevalence of Sex Chromosome Aneuploidy (SCA) Stratified by Genetic Ancestry The bars and error bars represent the calculated prevalence and 95% confidence interval (CI) for each genetic ancestry group. The horizontal line and shaded area represent the calculated prevalence and 95% CI overall. The lighter shaded areas within the bars represent individuals with a clinical diagnosis in their electronic medical record.

Of 595 612 genotyped males, we identified 862 with an additional X chromosome (47,XXY) and 747 with an extra Y chromosome (47,XYY), corresponding to a prevalence of 145 per 100 000 male population (1 in 690) for 47,XXY and 125 per 100 000 male population (1 in 800) for 47,XYY (Figure 1). The 862 males with 47,XXY were matched to 4310 controls and the 747 males with 47,XYY were matched to 3735 controls, resulting in a total study population of 9654 males. Of participants with 47,XXY, 87 were of African ancestry, 10 were of East Asian ancestry, 725 were of European ancestry, and 32 were of Hispanic ancestry. Of participants with 47,XYY, 46 were of African ancestry, 14 were of East Asian ancestry, 625 were of European ancestry, and 53 were of Hispanic ancestry (Table 1). The mean (SD) age at enrollment was 61.1 (12.0) years for participants with 47,XXY and 60.7 (12.2) years for participants with 47,XYY, similar to the overall MVP male population, at 61.5 (14.2) years (Table 1). However, genetic HARE was significantly different (Table 1; Figure 2). Estimated prevalence was more than twice as high in East Asian (47,XXY: 10 of 7313 participants; 47,XYY: 14 of 7313 participants) and European (47,XXY: 725 of 427 143 participants; 47,XYY: 625 of 427 143 participants) ancestry groups compared with African (47,XXY: 87 of 105 842 participants; 47,XYY: 46 of 105 842 participants) or Hispanic (47,XXY: 32 of 46 828 participants; 47,XYY: 53 of 46 828 participants) groups (*P* < .001). Among men with 47,XXY, 226 (26.2%) had a diagnosis in their EHR consistent with a clinical diagnosis of Klinefelter syndrome (KS), with a median (range) age of 52 (22-85) years at first diagnosis. Only 2 men (0.3%) with 47,XYY had a diagnosis in their EHR consistent with clinical ascertainment.

**Table 1.** Demographic Characteristics and Prevalence of Males With SCA Within the MVP Population

Characteristic	Individuals, No. (%)		
	All males in the MVP (N = 595 612)	Males with 47,XXY (n = 862)	Males with 47,XYY (n = 747)
Age at enrollment, y			
Mean (SD)	61.5 (14.2)	61.1 (12.0)	60.7 (12.2)
Median (range)	64 (18-106)	63 (22-91)	63 (26-97)
Genetic ancestry^a^^,^^b^			
African	105 842 (17.7)	87 (10.1)	46 (6.2)
East Asian	7313 (1.2)	10 (1.2)	14 (1.9)
European	427 143 (71.8)	725 (84.1)	625 (83.7)
Hispanic	46 828 (7.8)	32 (3.7)	53 (7.1)
Other or unavailable	8486 (1.4)	8 (0.9)	9 (1.2)
Clinical SCA diagnosis	228 (0.04)	226 (26.2)	2 (0.3)
Age first documented, median (range), y	52 (22-85)	52 (22-85)	59 (49-68)

Abbreviations: MVP, Million Veteran Program; SCA, sex chromosome aneuploidy.

^a^ Comparisons of ancestry categories for 47,XXY vs all MVP and 47,XYY vs all MVP are both *P* < .0001 based on χ^2^.

^b^ Genotype data within the GenISIS workspace were used to determine genetic ancestry using the harmonized ancestry and race and ethnicity method.

**Figure 3.**
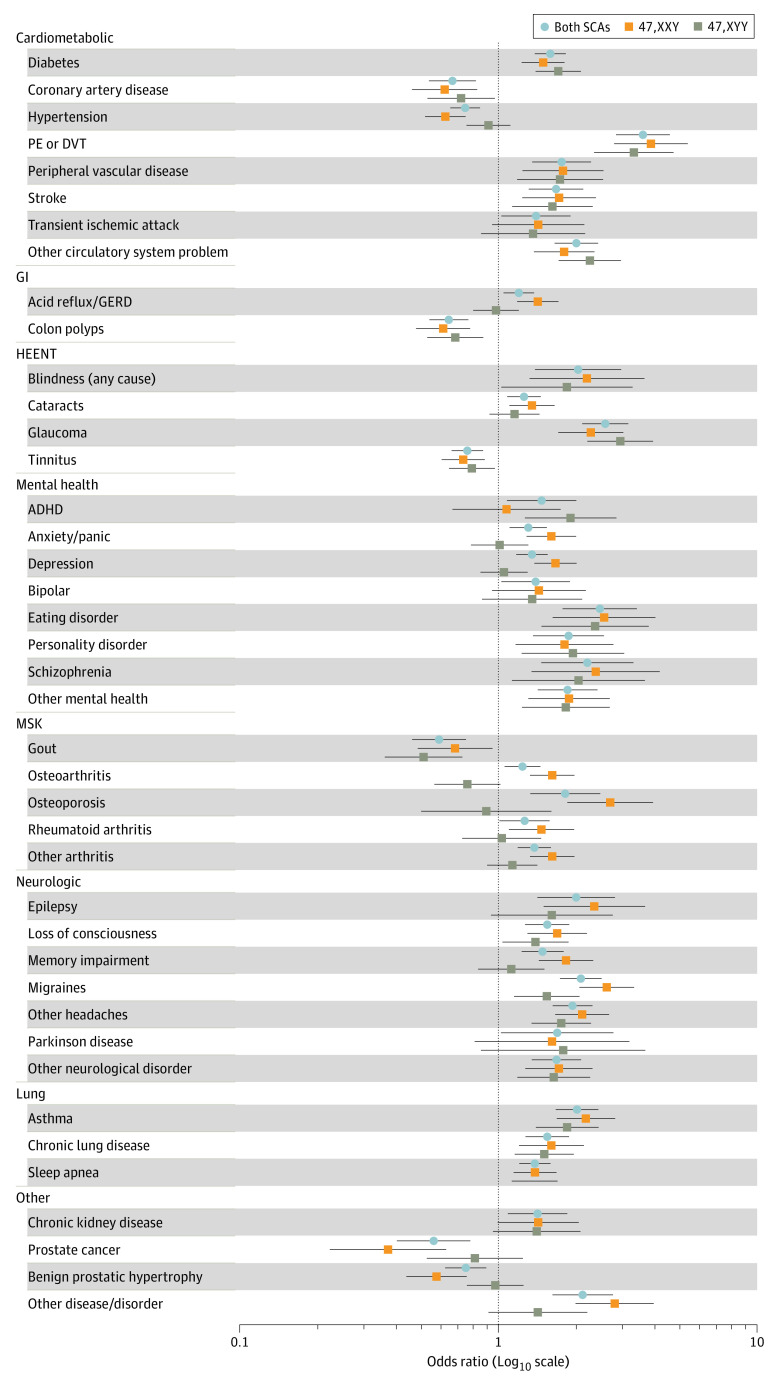
Self-Reported Diagnoses in Men With Sex Chromosome Aneuploidy (SCA) Odds of self-reported diagnoses are presented compared with their respective matched controls from the baseline survey. Point estimates are the odds ratios (OR) with odds greater than 1 representing higher odds in SCA; error bars represent the 95% CIs. Only conditions statistically different between SCA and controls and with a minimum of 10 individuals are included. ADHD indicates attention deficit/hyperactivity disorder; DVT, deep venous thrombosis; GERD, gastroesophageal reflux disease; GI, gastrointestinal; HEENT, head, eyes, ears, nose, throat; MSK, musculoskeletal; PE, pulmonary embolus.

After matching on age and ancestry, military service metrics were similar between SCA cases and controls and nearly all earned an Honorable Discharge (Table 2). Men with 47,XXY were 4.6 cm taller and men with 46,XYY were 9.2 cm taller than controls (*P* < .001). The CCI-calculated 10-year survival estimate mean was 44% (95% CI, 38%-50%) in men with 47,XXY (vs 56% [95% CI, 54%-58%] in controls; *P* < .001) and 39% (95% CI, 32%-46%) in men with 47,XYY (vs 59% [95% CI, 56%-61%] in controls; *P* < .001). The rates of outpatient, emergency, and inpatient encounters were 25% to 50% higher for SCA groups compared with controls (Table 2). For example the median (IQR) number of outpatient encounters per year was 22.6 (11.8-37.8) for 47,XXY vs 16.8 (9.4-28) for controls (*P* < .001) and 21.4 (12.4-33.8) for 47,XYY vs 17.0 (9.4-28.2) for controls (*P* < .001). Survey completion rates were similar in SCA and control groups (67% vs 64% for the baseline survey, and 48% for both groups for the lifestyle survey). Men with SCAs self-reported significantly more hospitalizations and prescription medications, along with lower health-related QOL lower (eg, mean [SD] self-reported physical function: 47,XXY: 34.2 [12] vs control mean [SD] 37.8 [12.8]; 47,XYY: 36.3 [11.6] vs control 37.9 [12.8]) (Table 2). Half of the 75 self-endorsed medical conditions were more common among men with SCAs compared with controls (Figure 3).

**Table 2.** Miliary Service, Morbidity, and Participant-Reported Outcome Measures Assessed From VHA Electronic Health Records and Baseline and Lifestyle Million Veteran Program Surveys

Measure	47,XXY Cohort			47,XYY Cohort		
	No. (%)		*P* value	No. (%)		*P* value
	Cases	Controls		Cases	Controls	
**Source: VHA records**						
No.	862	4310	NA	747	3735	NA
Period of miliary service						
Pre-Vietnam era	46 (5.3)	280 (6.5)	.12	48 (6.4)	248 (6.6)	.33
Vietnam era	463 (53.7)	2264 (52.8)		376 (50.3)	1882 (60.4)	
Post-Vietnam era	137 (15.9)	571 (13.3)		122 (16.3)	530 (14.2)	
Persian Gulf War era	163 (18.9)	918 (21.4)		148 (19.8)	839 (22.5)	
Other or missing	54 (6.1)	256 (6.0)		53 (7.1)	236 (6.3)	
First military service branch						
Air Force	59 (7.0)	334 (7.7)	.046	65 (8.7)	300 (8.0)	.53
Army	224 (26.6)	1189 (27.6)		194 (26.0)	996 (26.7)	
Navy	109 (12.9)	442 (10.2)		89 (11.9)	393 (10.5)	
Other^a^	50 (5.9)	346 (8.0)		51 (6.8)	308 (8.2)	
Unknown or missing	400 (47.5)	1999 (46.4)		348 (46.6)	1738 (46.5)	
Combat service	125 (14.5)	571 (13.2)	.71	102 (13.7)	512 (13.7)	.67
Military discharge status						
Honorable	422 (49.0)	2161 (50.1)	.40	361 (48.3)	1867 (50.0)	.01
General, Honorable Conditions	34 (3.9)	134 (3.1)		38 (5.1)	108 (2.3)	
Other than Honorable^b^	2 (0.2)	4 (0.1)		0 (0)	7 (0.2)	
Unknown or missing	404 (46.9)	2011 (46.7)		348 (46.6)	1753 (46.9)	
100% VHA service connection	121 (14.0)	668 (15.5)	.23	105 (14.1)	580 (15.5)	.33
Height at enrollment, mean (SD), cm	182.6 (7.8)	178.0 (7.1)	<.001	187.0 (8.3)	177.8 (7.3)	<.001
Weight at enrollment, mean (SD), kg	103.3 (23.0)	95.3 (20.2)	<.001	113.9 (28.3)	95.1 (20.2)	<.001
BMI at enrollment, mean (SD)	31.4 (6.6)	30.5 (6.1)	<.001	33.0 (7.7)	30.5 (6.1)	<.001
Duration of VHA data, median (IQR), y	18 (13-21)	17 (12-20)	<.001	17 (13-20)	16 (12-20)	<.001
Charlson Comorbidity Index						
Mean (SD)	4.30 (2.72)	3.90 (2.47)	<.001	4.45 (2.90)	3.82 (2.50)	<.001
10-y survival, % (95% CI)	44 (38-50)	56 (54-58)	<.001	39 (32-46)	59 (56-61)	<.001
Outpatient encounters, median (IQR), No. per y	22.6 (11.8-37.8)	16.8 (9.4-28.0)	<.001	21.4 (12.4-33.8)	17.0 (9.4-28.2)	<.001
Emergency encounters, median (IQR), No. per y	0.44 (0.19-1.0)	0.33 (0.14-0.7)	<.001	0.50 (0.20-0.95)	0.33 (0.14-0.68)	<.001
Inpatient encounters, median (IQR), No. per y	0.24 (0.11-0.49)	0.19 (0.1-0.42)	.001	0.24 (0.11-0.49)	0.19 (0.10-0.42)	.001
Deceased	110 (12.8)	510 (11.5)	.48	112 (15.0)	430 (11.5)	.02
Age of death, mean (SD), y	71.3 (9.6)	70.4 (9.5)	.39	71.3 (9.9)	71.4 (10.0)	.95
**Source: Baseline Survey**						
No.	574	2758	NA	500	2385	NA
Age at survey, mean (SD), y^c^	64.0 (10.0)	64.2 (10.2)	.72	63.7 (10.0)	63.5 (10.7)	.61
Military service branch(es)^d^						
Army	302 (52.6)	1366 (49.5)	.10	236 (47.2)	1152 (48.3)	.15
Navy	138 (24.0)	586 (21.2)		130 (26.0)	527 (22.1)	
Air Force	88 (15.3)	496 (18.0)		91 (18.2)	413 (17.3)	
Other^a^	101 (17.6)	562 (20.4)		88 (17.6)	499 (20.1)	
Deployed outside the US^e^	423 (73.7)	2076 (75.3)	.46	363 (72.6)	1773 (74.3)	.45
Exposure to biochemical warfare^e^	74 (12.9)	264 (9.6)	.04	64 (12.8)	230 (9.6)	.09
Health care from VHA facilities, %						
<50	156 (27.1)	813 (29.4)	.42	138 (27.6)	713 (29.9)	.16
51-75	49 (8.5)	189 (8.5)		42 (8.4)	158 (6.6)	
76-99	93 (16.2)	468 (16.2)		75 (15.0)	426 (17.9)	
Hospitalization this year^e^						
≥1 At VHA facility	162 (33.1)	484 (20.0)	<.001	110 (26.1)	413 (19.8)	.004
≥1 At non-VA facility	97 (25.5)	362 (18.1)	<.001	93 (26.4)	323 (18.6)	<.001
Prescription medications, No.						
0-3	180 (33.2)	1107 (42.9)	<.001	170 (36.2)	966 (43.3)	<.001
4-9	230 (42.4)	1068 (41.4)		194 (41.3)	914 (41.0)	
>10	132 (24.4)	407 (15.8)		106 (22.6)	348 (15.6)	
Health-related QOL^f^						
Physical functioning summary	34.2 (12.0)	37.8 (12.8)	<.001	36.3 (11.6)	37.9 (12.0)	.004
Mental health summary	45.3 (14.0)	47.8 (13.3)	<.001	46.3 (13.8)	47.3 (13.5)	.15
**Source: lifestyle survey**						
No.	429	2113	NA	350	1753	NA
Age at survey, mean (SD), y^c^	65.3 (9.7)	65.5 (9.4)	.70	64.6 (10.2)	64.8 (10.1)	.73
Reproductive health						
Sexual problems (eg, pain)	57 (13.5)	184 (8.9)	.005	30 (8.6)	157 (9.0)	.90
Erectile dysfunction	227 (53.9)	1218 (59.2)	.05	208 (59.4)	1025 (58.5)	.79
Fertility problems	215 (51.1)	139 (6.8)	<.001	53 (15.1)	121 (6.9)	<.001
Fathered a child	76 (18.1)	1627 (79.1)	<.001	200 (57.1)	1382 (78.8)	<.001
Neuropsychological health						
MOS Cog-R^g^	12.6 (7.4)	11.6 (7.1)	.01	13.1 (8.1)	11.7 (7.1)	.003
PHQ-4^h^						
Total	2.93 (3.47)	2.25 (3.17)	<.001	2.80 (3.26)	2.40 (3.26)	.046
Anxiety subscale	1.45 (1.13)	1.13 (1.69)	.001	1.38 (1.87)	1.21 (1.74)	.12
Depression subscale	1.54 (1.88)	1.15 (1.67)	<.001	1.46 (1.83)	1.20 (1.71)	.01
PCL total^i^	35.8 (17.1)	31.4 (15.6)	<.001	34.8 (15.8)	32.1 (12.9)	.004

Abbreviations: BMI, body mass index (calculated as weight in kilograms divided by height in meters squared); MOS Cog-R, Medical Outcomes Survey Cognitive Functioning Revised Scale; NA, not applicable; PCL, Posttraumatic Stress Disorder Checklist; PHQ-4, Patient Health Questionnaire-4; QOL, quality of life; VHA, Veterans Health Administration.

^a^ Other military branches include Coast Guard, Marine Corps, National Guard, and Merchant Marines.

^b^ Dishonorable Discharge was combined with Other than Honorable Discharge due to low numbers.

^c^ Participants were matched on age at time of study enrollment, which does not necessarily indicate balanced age at time of survey completion.

^d^ More than 1 selection was allowed; therefore, percentages add up to more than 100%.

^e^ At least 10% of respondents did not answer these questions; however, the proportion of nonresponders was similar among all groups, and results did not change if missing was included as a separate category.

^f^ Assessed using the Veterans RAND 12-Item Health Survey (range 0-100; higher values indicate better QOL).

^g^ Scores are norm-based, with higher values indicating more problems.

^h^ Total range, 0 to 12; subscale ranges, 0-6; higher values indicate more concern.

^i^ Range, 0 to 80; higher values indicate more concern.

Between enrollment and last follow-up, 110 individuals with 47,XXY (12.8%) and 112 individuals with 47,XYY (15.0%) died, compared with 510 (11.5%) and 430 (11.5%) age-matched controls, respectively (47,XXY: SMR, 1.1 [95% CI, 0.9-1.3]; 47,XYY: SMR, 1.3 [95% CI, 1.1-1.6]). Mean age of death did not differ (Table 2). Mortality was significantly greater for participants with an SCA compared with controls for leukemias (SMR, 10.0 [95% CI, 3.6-19.6]), diabetes (SMR, 2.5 [95% CI, 1.5-3.8), dementia (SMR, 2.5 [95% CI, 1.2-4.2]), and kidney disease (SMR, 3.0 [95% CI, 1.4-5.3]) (eTable 1 in Supplement 1).

Men with a clinical diagnosis of KS had a median of 4 more outpatient encounters per year and nearly double the number of emergency encounters per year compared with controls (eTable 2 in Supplement 1). Men with clinically diagnosed KS also reported a higher proportion of their care was received within the VHA system. Although more men with clinically diagnosed KS self-reported medical diagnoses (eFigure 2 in Supplement 1), summary measures of current mental health morbidity (Patient Health Questionnaire-4 and Posttraumatic Stress Disorder Checklist) were not different. CCI calculated from EHR data was 20% lower in men who were clinically diagnosed with KS and was no longer significantly different from their matched controls. Of the features classically ascribed to KS, self-reported diagnoses of breast cancer, osteoporosis, depression, anxiety, and bipolar disorder were more frequent in those with a clinical KS diagnosis, while height and self-reported infertility were not (Table 2; eFigure 2 and eTable 2 in Supplement 1). Other outcome measures were similar between men with 47,XXY with and without a clinical diagnosis of KS.

## Discussion

In this large, racially and ethnically diverse population-based cross-sectional study, we found a prevalence of 270 per 100 000 male population for X or Y chromosome aneuploidy among male veterans, with a novel difference in prevalence by genetic ancestry. While men with SCA experienced higher medical morbidity and lower health-related QOL with aging than nonaffected men, those with a clinical diagnosis of 47,XXY experienced lower medical comorbidity than those undiagnosed. These men successfully completed military service many years prior to study enrollment with similar service metrics to their 46,XY peers, suggesting that current Department of Defense codes preventing men with SCAs from military service should be reconsidered. This initial overview of men with 47,XXY and 47,XYY within the MVP highlights the variability of human disease and raises important racial, ethnic, and socioeconomic considerations for individuals participating in genetic research, particularly when such conditions may impact insurability and career trajectory.

Prior epidemiologic studies, including birth cohorts and several population-based cohorts, have estimated that 47,XXY occurs in 103 to 174 per 100 000 male population and 47,XYY in 69 to 98 individuals per 100 000 male population.^1,23^ Our estimates are within the expected range for 47,XXY (145 per 100 000 population) and higher than previously reported for 47,XYY (125 per 100 000 population). Few of these individuals had a clinical diagnosis in the EHR, and the age of diagnosis in this cohort was much older than previously reported, perhaps because individuals who were diagnosed earlier were prevented from serving in the US military.^24^

The ancestry-based difference in prevalence found in this study was unexpected because chromosomal aneuploidy is caused by random errors in meiosis and is assumed to be independent of genetic or social constructs.^25^ Our initial assumption was that parental age was a confounder, as this a known risk factor associated with nondisjunction errors, and parental age is higher in individuals with European ancestry compared with those with African or Hispanic ancestry.^26^ However, we noted a significant difference in parental age only between men with 47,XXY and controls and not between men with 47,XYY and controls; therefore, parental age did not explain the observed prevalence differences among genetic ancestry groups. Other nonrandom biological mechanisms contributing to nondisjunction may have a genetic or epigenetic basis with variations rooted in racial or ethnic ancestry.^27,28,29^ Alternatively, the cross-ancestry differences in prevalence could be secondary to sociodemographic factors, as enlistment in the US military requires conformation to a system of expectations that may be less attainable for minoritized individuals who also have physical, socioemotional, and learning differences that can be associated with SCAs.^30^ Further research into potential genetic, epigenetic, and/or sociodemographic factors is needed to understand the basis of the differences in SCA prevalence by ancestry that we observed in this population.

Our results corroborate findings from multiple other studies that associate SCAs with excess morbidity and higher health care utilization.^2,4,6^ Men with 47,XXY, independent of a known clinical diagnosis, were more likely than controls to endorse a history of numerous medical and psychological conditions, most of which have been previously reported and serve as external validation of our results.^2,3,4,5,6,7,8^ We also observed worse self-reported health and well-being metrics among men with SCA, which is congruent with current research, in which lower QOL outcomes are widely reported in individuals with SCAs.^8,31,32^ In contrast, we did not find greater mortality,^3,5,33^ perhaps because MVP does not capture deaths prior to enrollment; thus far, follow-up is relatively short, and the life expectancy of our controls was young (approximately 70 years), possibly reflecting worse health among US veterans.^34^

To our knowledge, this is the first study to compare morbidity, health care utilization, and health-related QOL between individuals with KS with and without a clinical diagnosis of KS. Although men with a clinical diagnosis of KS were neither taller nor more likely to report infertility than those without a clinical diagnosis, they were more likely to have a history of breast cancer, osteoporosis, and several mental health conditions previously associated with KS. Given the late diagnosis of KS (in the sixth decade of life), we speculate identification of these conditions led clinicians to suspect and test for KS, rather than vice versa. Finally, it is promising that a clinical diagnosis of KS, even if delayed, was associated with a lower CCI. This may potentially be due to closer medical follow-up and more preventive medicine interventions in the VHA, but additional studies are needed to determine whether the association between clinical diagnosis, increased outpatient encounters, and reduced morbidity is causal.

Biased stereotypes for individuals with SCAs^35^ can influence expectations and subsequent reporting for subjective measures. Our finding that QOL outcomes did not differ between those with and without a clinical diagnosis suggests a true association between SCA and QOL rather than clinical ascertainment as a confounder and is an important contribution to existing literature. In a 2018 study, health status was the most significant factor associated with QOL in men with 47,XXY,^31^ suggesting QOL may be increased if chronic health conditions are improved. In addition, many conditions contributing to morbidity in individuals with SCAs are preventable or at least treatable.^6,36^ We hypothesize that a more timely diagnosis could optimize medical care, thus leading to better QOL with aging.

With evidence of potentially preventable sequelae, SCAs exemplify the dilemma of whether, when, and how research participants should be notified of results from genetic research studies. Historically, the MVP does not return genetic results to participants, although a pilot is currently being conducted. Genetic results could have adverse implications for some participants, including potential denial of certain forms of insurance or employment opportunities.^37^ This is particularly true for veterans because they are not protected by the 2008 Genetic Information Nondiscrimination Act, which prohibits discrimination on the basis of genetic information with respect to health insurance and employment.^38^ Several cases from 1998 through 2017 have set a precedent that KS and sequelae associated with KS are preexisting conditions and excluded from military benefits.^39,40,41,42^ While a military service connection could be granted if service worsens the symptoms of a preexisting genetic disorder, we lack evidence about the effect of military service on the risk of disease penetrance among individuals with SCAs. The example of SCAs underscores the need for systems to enact safeguards for those considering clinical genetic testing or participating in genetic research without the threat of negative implications.

### Limitations

This study has some limitations. To our knowledge, this is the first ancestrally diverse investigation of SCAs, with rigorous cohort identification through genotyping that overcomes the clinical diagnostic bias inherent in most SCA studies to date. Despite these strengths, we recognized limitations in generalizing these findings to other populations. Individuals with a known SCA diagnosis and early comorbidities may have been dissuaded or ineligible from enrolling in the military, and clinicians within the VHA may be less likely to test for SCAs (reverse diagnostic suspicion bias), affecting the prevalence and diagnostic estimates within. We relied on the VHA EHR to identify clinical diagnoses of SCA, which may underestimate the true diagnostic rate if diagnoses obtained outside the VHA system were withheld. Similarly, the health care utilization metrics were obtained from the VHA EHR and do not capture encounters from outside facilities; however, the self-reported data did not suggest differences in VHA utilization. Furthermore, while the MVP cohort has provided a unique opportunity to study an aging population of men with SCAs, earlier outcomes may not be captured, and the immortal time bias may impact the SCA population eligible for enrollment and survey completion.^43^ Additionally, although the rate of survey response was congruent with the MVP cohort as a whole (67%), respondents may not be representative of the nonresponders with 47,XXY or 47,XYY. Recognizing these limitations, this robust study provides important, novel data for the SCA community and introduces the potential utility of this valuable resource.

## Conclusions

In this ancestrally diverse population-based cross-sectional study of men with 47,XXY or 47,XYY, we observed 270 of 100 000 men in the MVP sample had an extra X or Y chromosome, with an unexpected finding of a higher prevalence among European and East Asian ancestry groups. Our analysis found that men with SCA successfully complete military duty with similar service performance metrics as their 46,XY peers, and our findings suggest that a clinical diagnosis may reduce comorbidity burden. These data add to the existing literature on men with SCA with a favorable perspective on their contribution to society and highlight the need to study interventions that may ameliorate the negative health sequelae of SCA observed during aging.

## Notes

Supplement 1. **eFigure 1.** Scatterplot of Sex Chromosome Array Intensity **eFigure 2.** Self-Reported Medical History in Males with 47,XXY Stratified by Clinical Ascertainment Status **eTable 1.** Standardized Mortality Ratio by Cause of Death for Males with 47,XXY, 47,XYY, and Either Sex Chromosome Aneuploidy Compared With Matched 46,XY Controls **eTable 2.** Miliary Service, Morbidity, and Participant Reported Outcome Measures in Males With 47,XXY Stratified by Clinical Diagnosis Status **eTable 3.** Self-Reported Medical History in Males With Either Sex Chromosome Aneuploidy Compared to Matched 46,XY Controls **eTable 4.** Self-Reported Medical History in Males With 47,XXY Compared With Matched 46,XY Controls **eTable 5.** Self-Reported Medical History in Males With 47,XYY Compared With Matched 46,XY Controls

Supplement 2.
Data Sharing Statement
