# Supplementary material for: Prevalence, Morbidity, and Mortality of Men With Sex Chromosome Aneuploidy in the Million Veteran Program Cohort
Source: JAMA Netw Open. 2024 Mar 29;7(3):e244113. doi: 10.1001/jamanetworkopen.2024.4113 (PMC10980972; doi:10.1001/jamanetworkopen.2024.4113)
Supplement: Supplement 1. — eFigure 1. Scatterplot of Sex Chromosome Array Intensity eFigure 2. Self-Reported Medical History in Males with 47,XXY Stratified by Clinical Ascertainment Status eTable 1. Standardized Mortality Ratio by Cause of Death for Males with 47,XXY, 47,XYY, and Either Sex Chromosome Aneuploidy Compared With Matched 46,XY Controls eTable 2. Miliary Service, Morbidity, and Participant Reported Outcome Measures in Males With 47,XXY Stratified by Clinical Diagnosis Status eTable 3. Self-Reported Medical History in Males With Either Sex Chromosome Aneuploidy Compared to Matched 46,XY Controls eTable 4. Self-Reported Medical History in Males With 47,XXY Compared With Matched 46,XY Controls eTable 5. Self-Reported Medical History in Males With 47,XYY Compared With Matched 46,XY Controls [file jamanetwopen-e244113-s001.pdf]

Supplemental Online Content

Davis SM, Teerlink C, Lynch JA, et al. Prevalence, morbidity, and mortality of men with sex chromosome aneuploidy in the Million Veteran Program cohort. *JAMA Netw Open*. 2024;7(3):e244113. doi:10.1001/jamanetworkopen.2024.4113

eFigure 1. Scatterplot of Sex Chromosome Array Intensity
eFigure 2. Self-Reported Medical History in Males with 47,XXY Stratified by Clinical Ascertainment Status
eTable 1. Standardized Mortality Ratio by Cause of Death for Males with 47,XXY, 47,XYY, and Either Sex Chromosome Aneuploidy Compared With Matched 46,XY Controls
eTable 2. Miliary Service, Morbidity, and Participant Reported Outcome Measures in Males With 47,XXY Stratified by Clinical Diagnosis Status
eTable 3. Self-Reported Medical History in Males With Either Sex Chromosome Aneuploidy Compared to Matched 46,XY Controls
eTable 4. Self-Reported Medical History in Males With 47,XXY Compared With Matched 46,XY Controls
eTable 5. Self-Reported Medical History in Males With 47,XYY Compared With Matched 46,XY Controls

This supplemental material has been provided by the authors to give readers additional information about their work.

© 2024 Davis SM et al. *JAMA Network Open.*

Supplemental Figure 1. Scatterplot of sex chromosome array intensity over >20,000 X probes and 157 Y probes in all male MVP participants to identify men with an additional X or Y chromosome. Orange dots are males with 47,XXY, blue dots are males with 47,XYY, and black are males with 46,XY

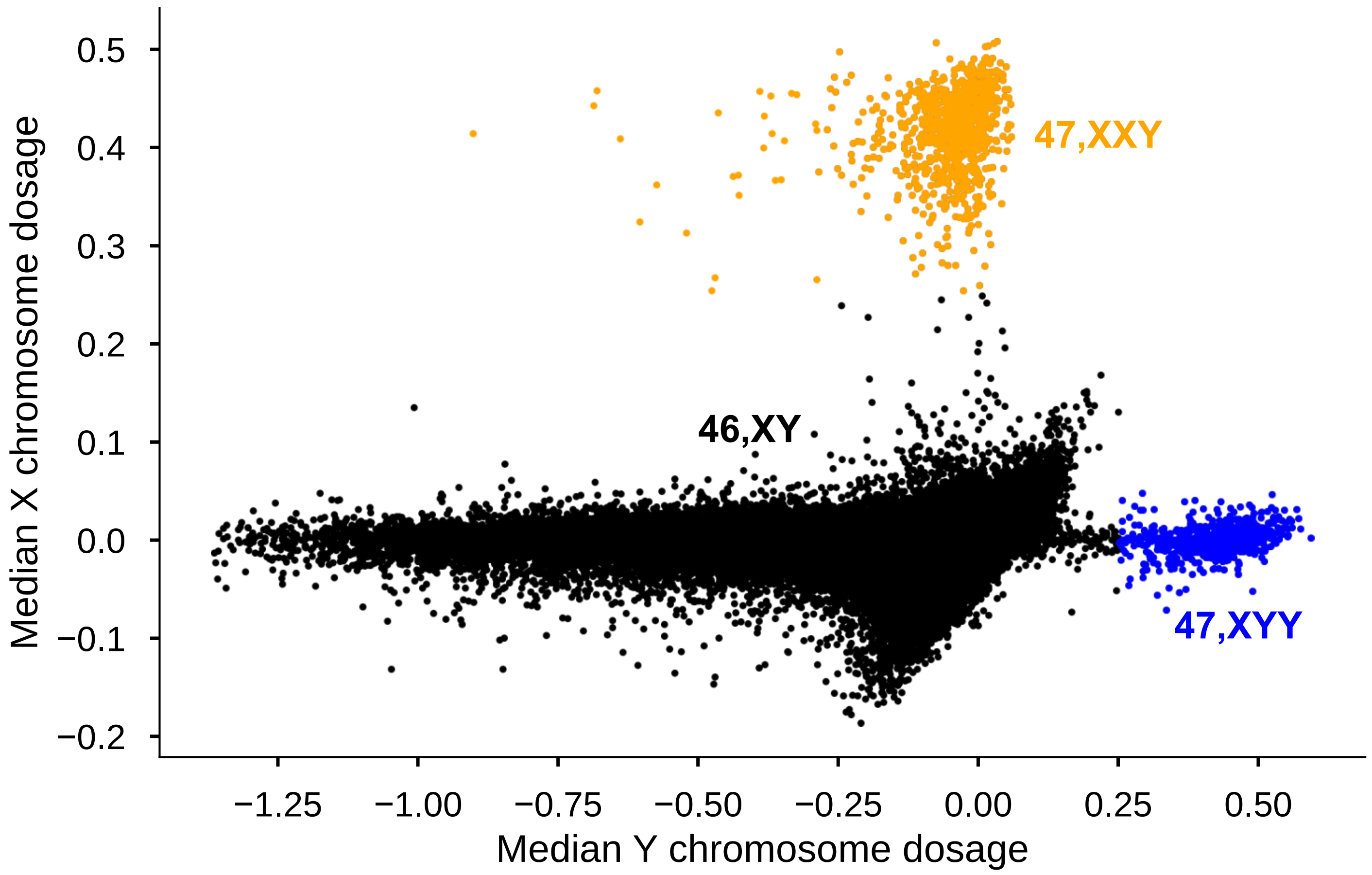

© 2024 Davis SM et al. *JAMA Network Open.*

Supplemental Figure 2. Self-Reported Medical History in Males with 47,XXY Stratified by Clinical Ascertainment Status
Forest plot with odds ratios (point estimate) and 95% confidence intervals (error bars) for self-reported conditions in men with 47,XXY versus 46,XY controls from the Baseline Survey. Only conditions that were significantly different ($p<0.05$) between men with 47,XXY and matched 46,XY controls (orange squares) are included. Individuals were then stratified into a clinical diagnosis of Klinefelter syndrome (gray circles) or no clinical diagnosis (white circles). Statistically significant differences in odds of reporting a specific medical condition between the diagnosed versus undiagnosed groups are indicated in the dashed rectangular boxes; gray indicates higher odds in those with a clinical diagnosis, red indicates higher odds in those without a clinical diagnosis.

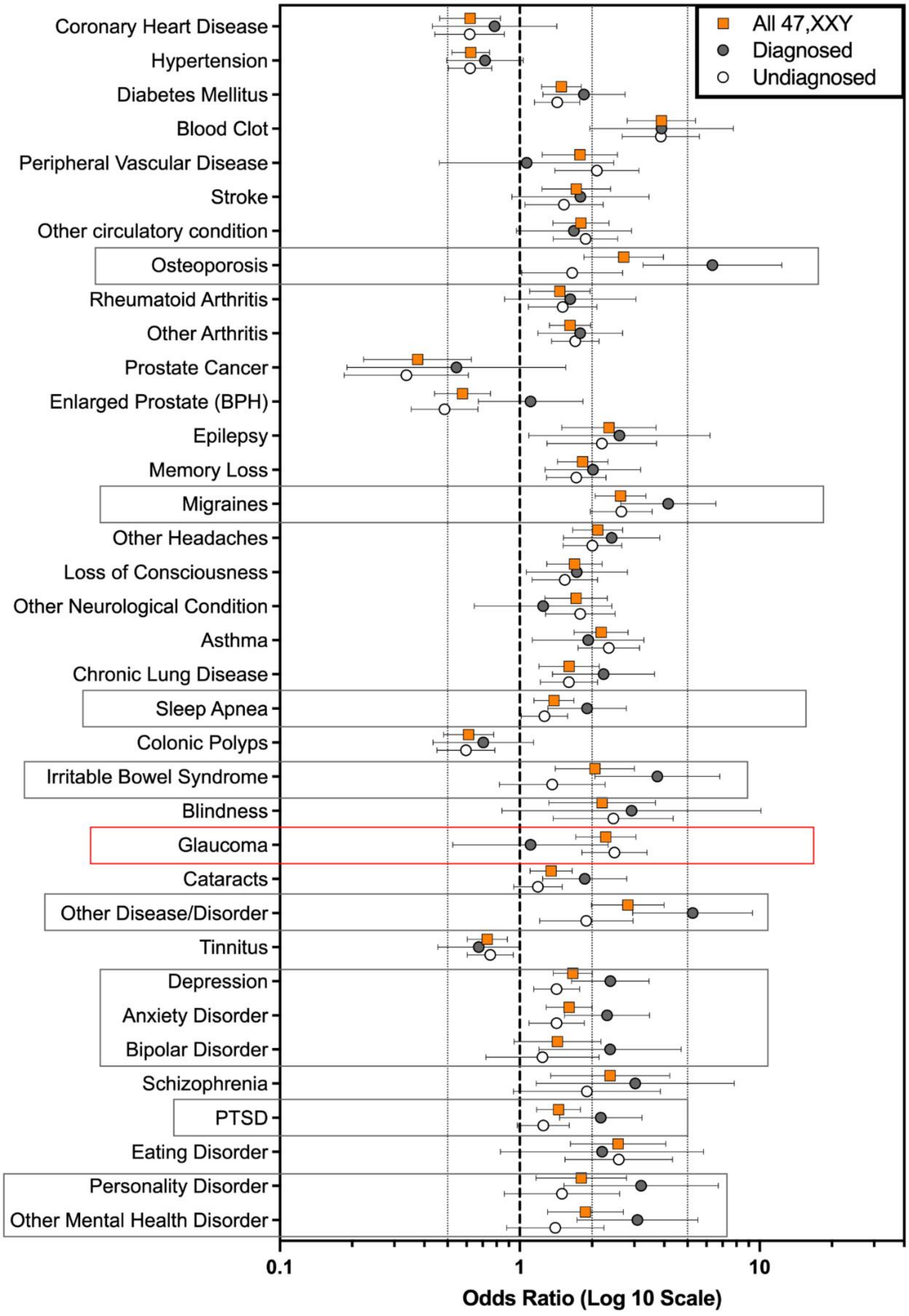

© 2024 Davis SM et al. *JAMA Network Open.*

Primary Cause of Death	47,XXY Only								47,XYY Only								All SCAs							
	Case Deaths		Control Deaths		Expected	SMR	LCI	UCI	Case Deaths		Control Deaths		Expected	SMR	LCI	UCI	Case Deaths		Control Deaths		Expected	SMR	LCI	UCI
	N	%	N	%	N				N	%	N	%	N				N	%	N	%	N			
All Causes	***110***	***100.0***	***510***	***100.0***	***102***	***1.08***	***0.89***	***1.29***	***112***	***100.0***	***430***	***100.0***	***86***	***1.30***	***1.07***	***1.55***	***222***	***100.0***	***940***	***100.0***	***188***	***1.18***	***1.03***	***1.34***
**Malignancies**	**28**	**25.5**	**170**	**33.3**	**34**	**0.82**	**0.55**	**1.16**	**23**	**20.5**	**153**	**35.6**	**30.6**	**0.75**	**0.48**	**1.09**	**51**	**23.0**	**323**	**34.4**	**64.6**	**0.79**	**0.59**	**1.02**
Leukemia	1	0.9	2	0.4	0.4	2.50	0.00	9.80	5	4.5	1	0.2	0.2	25.00	7.89	51.72	6	2.7	3	0.3	0.6	10.00	3.60	19.60
Non-Hodkin's lymphoma	1	0.9	3	0.6	0.6	1.67	0.00	6.53	1	0.9	3	0.7	0.6	1.67	0.00	6.53	2	0.9	6	0.6	1.2	1.67	0.16	4.78
Malignant neoplasm of colon, rectum & anus	2	1.8	10	2.0	2	1.00	0.09	2.87	1	0.9	10	2.3	2	0.50	0.00	1.96	3	1.4	20	2.1	4	0.75	0.14	1.84
Malignant neoplasm of pancreas	3	2.7	9	1.8	1.8	1.67	0.31	4.09	0	0.0	9	2.1	1.8				3	1.4	18	1.9	3.6	0.83	0.16	2.04
Malignant neoplasm of prostate	1	0.9	12	2.4	2.4	0.42	0.00	1.63	0	0.0	12	2.8	2.4				1	0.5	24	2.6	4.8	0.21	0.00	0.82
Malignant neoplasm of stomach	1	0.9	1	0.2	0.2	5.00	0.00	19.60	1	0.9	1	0.2	0.2	5.00	0.00	19.60	2	0.9	2	0.2	0.4	5.00	0.47	14.33
Malignant neoplasm of trachea, bronchus & lung	14	12.7	51	10.0	10.2	1.37	0.75	2.19	7	6.3	42	9.8	8.4	0.83	0.33	1.57	21	9.5	93	9.9	18.6	1.13	0.70	1.66
Malignant neoplasm of urinary tract	1	0.9	13	2.5	2.6	0.38	0.00	1.51	2	1.8	11	2.6	2.2	0.91	0.09	2.61	3	1.4	24	2.6	4.8	0.63	0.12	1.53
Other Malignant Neoplasms	4	3.6	69	13.5	13.8	0.29	0.08	0.64	6	5.4	64	14.9	12.8	0.47	0.17	0.92	10	4.5	133	14.1	26.6	0.38	0.18	0.65
**Cardiovascular Diseases**	**42**	**38.2**	**155**	**30.4**	**31**	**1.35**	**0.98**	**1.80**	**31**	**27.7**	**130**	**30.2**	**26**	**1.19**	**0.81**	**1.65**	**73**	**32.9**	**285**	**30.3**	**57**	**1.28**	**1.00**	**1.59**
Ischemic heart disease	11	10.0	76	14.9	15.2	0.72	0.36	1.21	15	13.4	57	13.3	11.4	1.32	0.73	2.07	26	11.7	133	14.1	26.6	0.98	0.64	1.39
Hypertension (incl hypertensive heart disease)	8	7.3	10	2.0	2	4.00	1.71	7.25	0	0.0	9	2.1	1.8				8	3.6	19	2.0	3.8	2.11	0.90	3.82
Other heart disease	12	10.9	36	7.1	7.2	1.67	0.86	2.74	7	6.3	31	7.2	6.2	1.13	0.45	2.12	19	8.6	67	7.1	13.4	1.42	0.85	2.13
Cerebrovascular Diseases	3	2.7	17	3.3	3.4	0.88	0.17	2.16	0	0.0	15	3.5	3				3	1.4	32	3.4	6.4	0.47	0.09	1.15
Diabetes Mellitus	8	7.3	16	3.1	3.2	2.50	1.07	4.53	9	8.0	18	4.2	3.6	2.50	1.13	4.40	17	7.7	34	3.6	6.8	2.50	1.45	3.83
**Other Diseases**	**32**	**29.1**	**149**	**29.2**	**29.8**	**1.07**	**0.73**	**1.48**	**46**	**41.1**	**117**	**27.2**	**23.4**	**1.97**	**1.44**	**2.57**	**78**	**35.1**	**266**	**28.3**	**53.2**	**1.47**	**1.16**	**1.81**
Dementia	4	3.6	12	2.4	2.4	1.67	0.43	3.70	7	6.3	10	2.3	2	3.50	1.39	6.57	11	5.0	22	2.3	4.4	2.50	1.24	4.20
Infections	3	2.7	17	3.3	3.4	0.88	0.17	2.16	6	5.4	12	2.8	2.4	2.50	0.90	4.90	9	4.1	29	3.1	5.8	1.55	0.70	2.73
Chronic Liver Disease	0	0.0	12	2.4	2.4				1	0.9	7	1.6	1.4	0.71	0.00	2.80	1	0.5	19	2.0	3.8	0.26	0.00	1.03
Nephritis, nephrotic syndrome and nephrosis	5	4.5	9	1.8	1.8	2.78	0.88	5.75	4	3.6	6	1.4	1.2	3.33	0.87	7.40	9	4.1	15	1.6	3	3.00	1.36	5.28
Chronic Lower Respiratory Diseases	8	7.3	37	7.3	7.4	1.08	0.46	1.96	8	7.1	31	7.2	6.2	1.29	0.55	2.34	16	7.2	68	7.2	13.6	1.18	0.67	1.82
Pneumonia	1	0.9	6	1.2	1.2	0.83	0.00	3.27	3	2.7	7	1.6	1.4	2.14	0.40	5.25	4	1.8	13	1.4	2.6	1.54	0.40	3.42
All other diseases	9	8.2	51	10.0	10.2	0.88	0.40	1.55	14	12.5	41	9.5	8.2	1.71	0.93	2.72	23	10.4	92	9.8	18.4	1.25	0.79	1.81
Symptoms, signs and abnormal findings NOS	2	1.8	5	1.0	1	2.00	0.19	5.73	3	2.7	3	0.7	0.6	5.00	0.94	12.26	5	2.3	8	0.9	1.6	3.13	0.99	6.46
**Trauma and Accidental**	**8**	**7.3**	**36**	**7.1**	**7.2**	**1.11**	**0.47**	**2.01**	**12**	**10.7**	**30**	**7.0**	**6**	**2.00**	**1.03**	**3.29**	**20**	**9.0**	**66**	**7.0**	**13.2**	**1.52**	**0.92**	**2.25**
Assault (Homicide)	0	0.0	1	0.2	0.2				0	0.0	1	0.2	0.2				0	0.0	2	0.2	0.4			
Intentional Self-Harm (Suicide)	1	0.9	9	1.8	1.8	0.56	0.00	2.18	3	2.7	7	1.6	1.4	2.14	0.40	5.25	4	1.8	16	1.7	3.2	1.25	0.33	2.78
Motor Vehicle Accidents	3	2.7	5	1.0	1	3.00	0.57	7.36	1	0.9	5	1.2	1	1.00	0.00	3.92	4	1.8	10	1.1	2	2.00	0.52	4.44
All other accidents and adverse effects	4	3.6	21	4.1	4.2	0.95	0.25	2.11	8	7.1	17	4.0	3.4	2.35	1.00	4.27	12	5.4	38	4.0	7.6	1.58	0.81	2.60

Table S1. Standardized Mortality Ratio by Cause of Death for Males with 47,XXY, 47,XYY, and Either Sex Chromosome Aneuploidy Compared to Matched 46,XY Controls. LCI = Lower 95% Confidence Interval of the SMR; UCI = Upper 95% Confidence interval of the SMR. NOS = not otherwise specified. Statistically significant differences are in red for increased risk and blue for decreased risk. SMR could not be calculated for causes without any deaths. Categories without any deaths are not included.

	Clinically Diagnosed Cohort			Undiagnosed Cohort			47,XXY diagnosed vs undiagnosed p-value
	Cases Total N=226 BL survey n=137 Life	Total N=1130 BL survey n=687 Life survey n=475	cases vs controls p-value	Cases Total N=640 BL survey n=437 Life survey	Total N=3200 BL survey n=2092 Life survey n=1560	cases vs controls p-value	
**Demographics and Military Service**							
Age at enrollment (yrs)	56.9 ± 13.4	56.9 ± 13.4	1	62.7 ± 11.2	62.7 ± 11.2	1	**<0.001**
European Genetic Ancestry	190 (84.1%)	950 (84.1%)	1	539 (84.2%)	2695 (84.2%)	1	0.958
Service Era: Vietnam	91 (40.2%)	456 (40.4%)	0.980	373 (58.3%)	1847 (57.7%)	0.793	**<0.001**
Military Branch: Army^	64 (50.0%)	312 (50.4%)	0.722	180 (53.9%)	869 (51.7%)	0.036	0.959
Combat Service: Yes^	45 (21.3%)	137 (14.5%)	**<0.001**	80 (12.5%)	410 (12.8%)	0.124	**0.005**
Deployed outside the US	96 (70.1%)	493 (71.8%)	0.680	329 (74.8%)	1585 (75.8%)	0.669	0.317
Exposure to biochemical warfare	24 (17.5%)	58 (8.4%)	**0.009**	50 (11.4%)	200 (9.6%)	0.420	0.200
Honorable Discharge^	120 (95.2%)	578 (93.8%)	0.776	303 (91.0%)	1566 (93.7%)	0.202	0.184
**Healthcare Utilization**							
Care within VA system: 100%	68 (49.6%)	289 (42.1%)	**0.004**	189 (43.0%)	912 (43.6%)	0.485	**0.009**
Outpatient encounters/yr	26.6 (14.9-43.2)	16.7 (9.3-28.0)	**<0.001**	22.2 (11.3-36.0)	17.2 (9.6-28.3)	**<0.001**	**0.001**
Emergency encounters/yr	0.46 (0.21-1.0)	0.37 (0.15-0.77)	**0.005**	0.26 (0.05-0.76)	0.33 (0.14-0.67)	**<0.001**	**<0.001**
Inpatient encounters/yr	0.11 (0.0-0.33)	0.05 (0.0-0.24)	**<0.001**	0.13 (0.0-0.33)	0.06 (0.0-0.24)	**<0.001**	0.455
Hospitalized in past year (VA)	40 (29.2%)	113 (16.5%)	**<0.001**	122 (27.9%)	359 (17.2%)	**<0.001**	0.642
**Medical Characteristics & Morbidity**							
Height (cm) at enrollment	182.7 ± 8.4	178.4 ± 7.0	**<0.001**	182.5 ± 7.7	178.0 ± 7.2	**<0.001**	0.822
Weight (kg) at enrollment	103.3 ± 21.9	96.1 ± 20.1	**<0.001**	103.1 ± 23.4	94.9 ± 20.1	**<0.001**	0.902
BMI (kg/m2) at enrollment	31.4 ± 6.2	30.8 ± 6.2	0.153	31.4 ± 6.8	30.4 ± 6.1	**<0.001**	0.954
Charlson Comorbidity Index	3.65 ± 2.73	3.39 ± 2.55	0.198	4.53 ± 2.68	4.13 ± 2.47	**<0.001**	**<0.001**
>10 Prescription Medications	35 (25.5%)	95 (13.8%)	**<0.001**	97 (22.0%)	311 (14.9%)	**<0.001**	0.052
Sexual Health Problems	18 (20.5%)	40 (8.4%)	**0.002**	39 (11.6%)	152 (9.7%)	0.272	**0.036**
Fertility Problems	52 (59.1%)	31 (6.5%)	**<0.001**	164 (48.8%)	110 (7.1%)	**<0.001**	0.094
**Psychosocial Morbidity**							
VR-12 Physical Functioning	**34.8 ± 12.4**	**38.4 ± 13.3**	**0.005**	**34.0 ± 11.9**	**37.6 ± 12.8**	**<0.001**	0.519
VR-12 Mental Health	**42.8 ± 15.0**	**46.5 ± 14.0**	**0.009**	**46.0 ± 13.6**	**48.2 ± 13.2**	**0.003**	**0.023**
MOS Cog-R	12.6 ± 6.0	12.2 ± 7.6	0.608	12.6 ± 7.7	11.5 ± 7.1	**0.020**	0.985
PHQ4 Total	3.14 ± 3.4	2.45 ± 3.29	0.084	2.87 ± 3.5	2.23 ± 3.2	**0.002**	0.487
PHQ4 Anxiety Subscale	1.54 ± 1.87	1.19 ± 1.7	0.106	1.42 ± 1.86	1.12 ± 1.7	**0.005**	0.598
PHQ4 Depression Subscale	1.6 ± 1.78	1.29 ± 1.78	0.128	1.51 ± 1.91	1.14 ± 1.68	**<0.001**	0.657
PCL Total	36.5 ± 17.0	32.6 ± 16.3	0.055	35.6 ± 17.1	31.5 ± 15.7	**<0.001**	0.649
**Mortality**							
Deceased	20 (8.8%)	127 (11.2%)	0.292	90 (14.1%)	410 (12.8%)	0.493	0.092

Table S2. Miliary Service, Morbidity, and Participant Reported Outcome Measures in Males with 47,XXY Stratified by Clinical Diagnosis Status. Colored text indicate the data source and subsequently the sample size available for that variable: black is the electronic health record database, blue is the Baseline Survey, and green is the Lifestyle Survey. Data are shown as mean ± standard deviation (SD) and/or median (range) for continuous data, and n (%) for categorical data, unless otherwise specified. Bolded p-values indicate statistical significance at an alpha of 0.05. ^Percentages are calculated from the total with non-missing data; chi-squared analyses included missing as a category. VA = Veterans Affairs; VR-12 = Veterans RAND 12 Item Health Survey (higher values indicate better QoL). MOS Cog-R = Medical Outcomes Survey Cognitive Functioning Revised Scale (higher values indicate more problems). PHQ-4 = Patient Health Questionnaire-4 (higher values indicate more concern). PCL = Post-traumatic stress disorder Checklist (higher values indicate more concern).

© 2024 Davis SM et al. *JAMA Network Open.*

Table S3a. Self-Reported Medical History in Males with Either Sex Chromosome Aneuploidy Compared to Matched 46,XY Controls							
	Any SCA		Controls				
	with	w/o	with	w/o	OR	U95%CI	L95%CI
Circ-Congestive Heart Failure	69	1005	303	4840	1.10	1.44	0.84
**Circ-Coronary Artery Disease**	**114**	**960**	**779**	**4364**	**0.67**	**0.82**	**0.54**
Circ-High Cholesterol	555	519	2604	2539	1.04	1.19	0.91
**Circ-Hypertension**	**584**	**490**	**3164**	**1979**	**0.75**	**0.85**	**0.65**
Circ-Myocardial Infarction	111	963	612	4531	0.85	1.06	0.69
**Circ-Other Circ System Problem**	**165**	**909**	**427**	**4716**	**2.00**	**2.43**	**1.65**
**Circ-PE or DVT**	**123**	**951**	**177**	**4966**	**3.63**	**4.61**	**2.85**
**Circ-Peripheral Vascular Disease**	**81**	**993**	**228**	**4915**	**1.76**	**2.29**	**1.35**
**Circ-Stroke**	**95**	**979**	**282**	**4861**	**1.67**	**2.13**	**1.31**
**Circ-Transient Ischemic Attack**	**55**	**1019**	**191**	**4952**	**1.40**	**1.90**	**1.03**
**Endo-Diabetes**	**401**	**673**	**1403**	**3740**	**1.59**	**1.82**	**1.38**
Endo-Thyroid problem	88	986	434	4709	0.97	1.23	0.76
**GI-Acid Reflux/GERD**	**401**	**673**	**1764**	**3553**	**1.20**	**1.38**	**1.05**
**GI-Bowel obstruction**	**29**	**1045**	**76**	**5067**	**1.85**	**2.85**	**1.20**
GI-Celiac disease	3	1071	10	5133	1.44	5.23	0.40
**GI-Colon Polyps**	**178**	**896**	**1212**	**3931**	**0.64**	**0.77**	**0.54**
GI-Crohn's Disease	7	1067	26	5117	1.29	2.98	0.56
**GI-Irritable Bowel Syndrome**	**45**	**1029**	**139**	**5004**	**1.57**	**2.22**	**1.12**
GI-Liver condition (eg cirrhosis)	41	1033	190	4953	1.03	1.46	0.73
GI-Other	67	1007	263	4880	1.23	1.63	0.94
GI-Peptic Ulcers	50	1024	188	4955	1.29	1.77	0.94
GI-Ulcerative colitis	13	1061	61	5082	1.02	1.86	0.56
**HEENT-Blindness-any cause**	**38**	**1036**	**91**	**5052**	**2.04**	**2.99**	**1.39**
**HEENT-Cataracts**	**292**	**782**	**1177**	**3966**	**1.26**	**1.46**	**1.08**
HEENT-Deafness (partial/severe)	340	734	1556	3587	1.07	1.23	0.93
**HEENT-Glaucoma**	**154**	**920**	**312**	**4831**	**2.59**	**3.18**	**2.11**
HEENT-Macular Degeneration	50	1024	233	4910	1.03	1.41	0.75
**HEENT-Tinnitus**	**343**	**731**	**1965**	**3178**	**0.76**	**0.87**	**0.66**
Infx-Hepatitis C	52	1022	284	4859	0.87	1.18	0.64
Infx-HIV/AIDS	12	1062	54	5089	1.06	2.00	0.57
Infx-Other	37	1037	124	5019	1.44	2.10	0.99
Infx-Tuberculosis	22	1052	90	5053	1.17	1.88	0.73
**MH-ADHD**	**55**	**1019**	**182**	**4961**	**1.47**	**2.00**	**1.08**
**MH-Anxiety/Panic**	**217**	**857**	**835**	**4308**	**1.31**	**1.54**	**1.11**
**MH-Bipolar**	**56**	**1018**	**195**	**4948**	**1.40**	**1.89**	**1.03**
**MH-Depression**	**374**	**700**	**1458**	**3685**	**1.35**	**1.55**	**1.17**
**MH-Eating disorder**	**55**	**1019**	**110**	**5033**	**2.47**	**3.44**	**1.77**

© 2024 Davis SM et al. *JAMA Network Open.*

**MH-Other**	**80**	**994**	**214**	**4929**	**1.85**	**2.42**	**1.42**
**MH-Personality disorder**	**56**	**1018**	**147**	**4996**	**1.87**	**2.56**	**1.36**
MH-PTSD	194	880	812	4331	1.18	1.40	0.99
**MH-Schizophrenia**	**34**	**1040**	**75**	**5068**	**2.21**	**3.33**	**1.47**
MH-Social Phobia	33	1041	175	4968	0.90	1.31	0.62
**MSK-Gout**	**82**	**992**	**644**	**4599**	**0.59**	**0.75**	**0.46**
**MSK-Osteoarthritis**	**240**	**834**	**968**	**4175**	**1.24**	**1.46**	**1.06**
**MSK-Osteoporosis**	**57**	**1017**	**154**	**4989**	**1.82**	**2.48**	**1.33**
**MSK-Other arthritis**	**306**	**768**	**1153**	**3990**	**1.38**	**1.60**	**1.19**
MSK-Other MSK problem	217	857	1114	4029	0.92	1.08	0.78
**MSK-Rheumatoid arthritis**	**107**	**967**	**414**	**4729**	**1.26**	**1.58**	**1.01**
Neuro-ALS	2	1072	3	5140	3.20	19.15	0.53
Neuro-Dementia	16	1058	50	5093	1.54	2.72	0.87
**Neuro-Epilepsy**	**47**	**1027**	**115**	**5028**	**2.00**	**2.83**	**1.42**
**Neuro-Loss of consciousness**	**149**	**925**	**485**	**4658**	**1.55**	**1.88**	**1.27**
**Neuro-Memory loss/impairment**	**168**	**906**	**571**	**4572**	**1.48**	**1.79**	**1.23**
**Neuro-Migraines**	**183**	**891**	**461**	**4682**	**2.09**	**2.51**	**1.73**
Neuro-MS	4	1070	27	5116	0.71	2.03	0.25
**Neuro-Other**	**118**	**956**	**352**	**4791**	**1.68**	**2.09**	**1.35**
**Neuro-Other Headaches**	**199**	**875**	**540**	**4603**	**1.94**	**2.32**	**1.62**
**Neuro-Parkinson's disease**	**21**	**1053**	**60**	**5083**	**1.69**	**2.79**	**1.02**
Neuro-Spinal Cord Injury	95	979	389	4754	1.19	1.50	0.94
Neuro-Traumatic Brain Injury	48	1026	199	4944	1.16	1.60	0.84
**Onc-Breast cancer**	**6**	**1068**	**5**	**5138**	**5.77**	**18.95**	**1.76**
Onc-Colorectal cancer	16	1058	86	5057	0.89	1.52	0.52
Onc-Lung cancer	16	1058	61	5082	1.26	2.19	0.72
Onc-other cancer	74	1000	366	4777	0.97	1.25	0.75
**Onc-Prostate cancer**	**42**	**1032**	**347**	**4796**	**0.56**	**0.78**	**0.41**
Onc-Skin cancer	137	937	742	4401	0.87	1.05	0.71
**Other disease/disorder**	**80**	**994**	**188**	**4955**	**2.12**	**2.78**	**1.62**
**Other-Benign Prostatic Hypertrophy**	**157**	**917**	**957**	**4186**	**0.75**	**0.90**	**0.62**
**Pulm-Asthma**	**172**	**902**	**444**	**4699**	**2.02**	**2.44**	**1.67**
**Pulm-Chronic lung disease**	**154**	**920**	**502**	**4641**	**1.55**	**1.88**	**1.27**
**Pulm-Sleep apnea**	**373**	**701**	**1427**	**3716**	**1.39**	**1.59**	**1.21**
Renal-Acute Kidney Disease	20	1054	63	5080	1.53	2.54	0.92
**Renal-Kidney Disease no dialysis**	**76**	**998**	**262**	**4881**	**1.42**	**1.85**	**1.09**
**Renal-Kidney Disease with dialysis**	**21**	**1053**	**61**	**5082**	**1.66**	**2.74**	**1.01**
Skin Condition	119	955	520	4623	1.11	1.37	0.90

The number of participants endorsing each of the 75 potential conditions from the Baseline Survey and calculated odds ratio (OR) with upper and lower 95% confidence intervals (U95%CI, L95CI). Bolded values are significantly different between SCA cases and controls at an alpha of 0.05

© 2024 Davis SM et al. *JAMA Network Open.*

**Table S3b. Self-Reported Medical History in Males with 47,XXY Compared to Matched 46,XY Controls**							
	**47,XXY**		**Controls**				
	**with**	**w/o**	**with**	**w/o**	**OR**	**U95%CI**	**L95%CI**
Circ-Congestive Heart Failure	40	534	167	2591	1.16	1.66	0.81
**Circ-Coronary Artery Disease**	**58**	**516**	**423**	**2335**	**0.62**	**0.83**	**0.46**
Circ-High Cholesterol	276	298	1308	1450	1.03	1.23	0.86
**Circ-Hypertension**	**291**	**283**	**1716**	**1042**	**0.62**	**0.75**	**0.52**
Circ-Myocardial Infarction	61	513	333	2425	0.87	1.16	0.65
**Circ-Other Circ System Problem**	**82**	**492**	**234**	**2524**	**1.80**	**2.35**	**1.37**
**Circ-PE or DVT**	**68**	**506**	**92**	**2666**	**3.89**	**5.40**	**2.81**
**Circ-Peripheral Vascular Disease**	**43**	**531**	**120**	**2638**	**1.78**	**2.55**	**1.24**
**Circ-Stroke**	**52**	**522**	**151**	**2607**	**1.72**	**2.39**	**1.24**
Circ-Transient Ischemic Attack	31	543	106	2652	1.43	2.15	0.95
**Endo-Diabetes**	**206**	**368**	**753**	**2005**	**1.49**	**1.80**	**1.23**
Endo-Thyroid problem	49	525	237	2521	0.99	1.37	0.72
**GI-Acid Reflux/GERD**	**232**	**342**	**947**	**1985**	**1.42**	**1.71**	**1.18**
GI-Bowel obstruction	9	565	25	2733	1.74	3.75	0.81
GI-Celiac disease	1	573	5	2753	0.96	8.24	0.11
**GI-Colon Polyps**	**91**	**483**	**649**	**2109**	**0.61**	**0.78**	**0.48**
GI-Crohn's Disease	6	568	11	2747	2.64	7.16	0.97
**GI-Irritable Bowel Syndrome**	**40**	**534**	**97**	**2661**	**2.05**	**3.00**	**1.41**
GI-Liver condition (eg cirrhosis)	20	554	100	2658	0.96	1.56	0.59
GI-Other	39	535	141	2617	1.35	1.95	0.94
GI-Peptic Ulcers	19	555	97	2661	0.94	1.55	0.57
GI-Ulcerative colitis	7	567	33	2725	1.02	2.32	0.45
**HEENT-Blindness-any cause**	**22**	**552**	**49**	**2709**	**2.20**	**3.67**	**1.32**
**HEENT-Cataracts**	**164**	**410**	**630**	**2128**	**1.35**	**1.65**	**1.10**
HEENT-Deafness (partial/severe)	158	416	844	1914	0.86	1.05	0.70
**HEENT-Glaucoma**	**74**	**500**	**168**	**2590**	**2.28**	**3.05**	**1.71**
HEENT-Macular Degeneration	22	552	122	2636	0.86	1.37	0.54
**HEENT-Tinnitus**	**180**	**394**	**1060**	**1698**	**0.73**	**0.89**	**0.60**
Infx-Hepatitis C	25	549	156	2602	0.76	1.17	0.49
Infx-HIV/AIDS	5	569	28	2730	0.86	2.23	0.33
Infx-Other	22	552	66	2692	1.63	2.66	0.99
Infx-Tuberculosis	12	562	46	2712	1.26	2.39	0.66
**MH-ADHD**	**21**	**553**	**94**	**2664**	**1.08**	**1.74**	**0.66**
**MH-Anxiety/Panic**	**131**	**443**	**429**	**2329**	**1.61**	**2.00**	**1.29**
MH-Bipolar	30	544	102	2656	1.44	2.18	0.95
**MH-Depression**	**221**	**353**	**754**	**2004**	**1.66**	**2.01**	**1.38**
**MH-Eating disorder**	**29**	**545**	**56**	**2702**	**2.57**	**4.06**	**1.62**
**MH-Other**	**43**	**531**	**114**	**2644**	**1.88**	**2.70**	**1.31**

© 2024 Davis SM et al. *JAMA Network Open.*

**MH-Personality disorder**	**29**	**545**	**79**	**2679**	**1.80**	**2.79**	**1.17**
**MH-PTSD**	**148**	**426**	**533**	**2225**	**1.45**	**1.79**	**1.18**
**MH-Schizophrenia**	**18**	**556**	**37**	**2721**	**2.38**	**4.21**	**1.35**
MH-Social Phobia	17	557	91	2667	0.89	1.51	0.53
**MSK-Gout**	**43**	**531**	**293**	**2465**	**0.68**	**0.95**	**0.49**
**MSK-Osteoarthritis**	**181**	**393**	**611**	**2147**	**1.62**	**1.97**	**1.33**
**MSK-Osteoporosis**	**43**	**531**	**80**	**2678**	**2.71**	**3.97**	**1.85**
**MSK-Other arthritis**	**181**	**393**	**611**	**2147**	**1.62**	**1.97**	**1.33**
MSK-Other MSK problem	126	448	584	2174	1.05	1.30	0.84
**MSK-Rheumatoid arthritis**	**66**	**508**	**224**	**2534**	**1.47**	**1.97**	**1.10**
Neuro-ALS	0	574	2	2756			
Neuro-Dementia	10	564	27	2731	1.79	3.73	0.86
**Neuro-Epilepsy**	**29**	**545**	**61**	**2697**	**2.35**	**3.70**	**1.50**
**Neuro-Loss of consciousness**	**84**	**490**	**254**	**2504**	**1.69**	**2.20**	**1.30**
**Neuro-Memory loss/impairment**	**106**	**468**	**304**	**2454**	**1.83**	**2.33**	**1.44**
**Neuro-Migraines**	**115**	**459**	**240**	**2518**	**2.63**	**3.35**	**2.06**
Neuro-MS	2	572	15	2743	0.64	2.80	0.15
**Neuro-Other**	**64**	**510**	**188**	**2570**	**1.72**	**2.31**	**1.27**
**Neuro-Other Headaches**	**112**	**462**	**284**	**2474**	**2.11**	**2.69**	**1.66**
Neuro-Parkinson's disease	11	563	33	2725	1.61	3.21	0.81
Neuro-Spinal Cord Injury	50	524	205	2553	1.19	1.64	0.86
Neuro-Traumatic Brain Injury	27	547	108	2650	1.21	1.86	0.79
**Onc-Breast cancer**	**6**	**568**	**3**	**2755**	**9.70**	**38.90**	**2.42**
Onc-Colorectal cancer	8	566	46	2712	0.83	1.78	0.39
Onc-Lung cancer	7	567	32	2726	1.05	2.39	0.46
Onc-other cancer	40	534	193	2565	1.00	1.42	0.70
**Onc-Prostate cancer**	**16**	**558**	**196**	**2562**	**0.37**	**0.63**	**0.22**
Onc-Skin cancer	69	505	386	2372	0.84	1.10	0.64
**Other disease/disorder**	**53**	**521**	**96**	**2662**	**2.82**	**4.00**	**1.99**
**Other-Benign Prostatic Hypertrophy**	**69**	**505**	**528**	**2230**	**0.58**	**0.76**	**0.44**
**Pulm-Asthma**	**94**	**480**	**227**	**2531**	**2.18**	**2.83**	**1.68**
**Pulm-Chronic lung disease**	**68**	**506**	**213**	**2545**	**1.61**	**2.14**	**1.20**
**Pulm-Sleep apnea**	**199**	**375**	**763**	**1995**	**1.39**	**1.68**	**1.15**
Renal-Acute Kidney Disease	8	566	36	2722	1.07	2.31	0.49
Renal-Kidney Disease no dialysis	41	533	141	2617	1.43	2.05	1.00
Renal-Kidney Disease with dialysis	9	565	32	2726	1.36	2.86	0.64
Skin Condition	60	514	277	2481	1.05	1.40	0.78

The number of participants endorsing each of the 75 potential conditions from the Baseline Survey and calculated odds ratio (OR) with upper and lower 95% confidence intervals (U95%CI, L95CI). Bolded values are significantly different between SCA cases and controls at an alpha of 0.05

© 2024 Davis SM et al. *JAMA Network Open.*

Table S3c. Self-Reported Medical History in Males with 47,XYY Compared to Matched 46,XY Controls							
	47,XYY		Controls				
	with	w/o	with	w/o	OR	U95%CI	L95%CI
Circ-Congestive Heart Failure	29	471	136	2249	1.02	1.54	0.67
**Circ-Coronary Artery Disease**	**56**	**444**	**356**	**2029**	**0.72**	**0.97**	**0.53**
Circ-High Cholesterol	279	221	1296	1089	1.06	1.29	0.87
Circ-Hypertension	293	207	1448	937	0.92	1.11	0.75
Circ-Myocardial Infarction	50	450	279	2106	0.84	1.15	0.61
**Circ-Other Circ System Problem**	**83**	**417**	**193**	**2192**	**2.26**	**2.98**	**1.71**
**Circ-PE or DVT**	**55**	**445**	**85**	**2300**	**3.34**	**4.77**	**2.35**
**Circ-Peripheral Vascular Disease**	**38**	**462**	**108**	**2277**	**1.73**	**2.54**	**1.18**
**Circ-Stroke**	**43**	**457**	**131**	**2254**	**1.62**	**2.32**	**1.13**
Circ-Transient Ischemic Attack	24	476	85	2300	1.36	2.17	0.86
**Endo-Diabetes**	**195**	**305**	**650**	**1735**	**1.71**	**2.09**	**1.40**
Endo-Thyroid problem	39	461	197	2188	0.94	1.34	0.66
GI-Acid Reflux/GERD	169	331	817	1568	0.98	1.20	0.80
**GI-Bowel obstruction**	**20**	**480**	**51**	**2334**	**1.91**	**3.23**	**1.13**
GI-Celiac disease	2	498	5	2380	1.91	9.88	0.37
**GI-Colon Polyps**	**87**	**413**	**563**	**1822**	**0.68**	**0.88**	**0.53**
GI-Crohn's Disease	1	499	15	2370	0.32	2.40	0.04
GI-Irritable Bowel Syndrome	5	495	42	2343	0.56	1.43	0.22
GI-Liver condition (eg cirrhosis)	21	479	90	2295	1.12	1.82	0.69
GI-Other	28	472	122	2263	1.10	1.68	0.72
**GI-Peptic Ulcers**	**31**	**469**	**91**	**2294**	**1.67**	**2.53**	**1.10**
GI-Ulcerative colitis	6	494	28	2357	1.02	2.48	0.42
**HEENT-Blindness-any cause**	**16**	**484**	**42**	**2343**	**1.84**	**3.31**	**1.03**
HEENT-Cataracts	128	372	547	1838	1.16	1.44	0.93
**HEENT-Deafness (partial/severe)**	**182**	**318**	**712**	**1673**	**1.34**	**1.65**	**1.10**
**HEENT-Glaucoma**	**80**	**420**	**144**	**2241**	**2.96**	**3.97**	**2.21**
HEENT-Macular Degeneration	28	472	111	2274	1.22	1.86	0.79
**HEENT-Tinnitus**	**163**	**337**	**905**	**1480**	**0.79**	**0.97**	**0.64**
Infx-Hepatitis C	27	473	128	2257	1.01	1.54	0.66
Infx-HIV/AIDS	7	493	26	2359	1.29	2.98	0.56
Infx-Other	15	485	58	2327	1.24	2.21	0.70
Infx-Tuberculosis	10	490	44	2341	1.09	2.17	0.54
**MH-ADHD**	**34**	**466**	**88**	**2297**	**1.90**	**2.86**	**1.27**
MH-Anxiety/Panic	86	414	406	1979	1.01	1.31	0.78
MH-Bipolar	26	474	93	2292	1.35	2.11	0.87
MH-Depression	153	347	704	1681	1.05	1.30	0.85
**MH-Eating disorder**	**26**	**474**	**54**	**2331**	**2.37**	**3.82**	**1.47**
**MH-Other**	**37**	**463**	**100**	**2285**	**1.83**	**2.70**	**1.24**
**MH-Personality disorder**	**27**	**473**	**68**	**2317**	**1.95**	**3.07**	**1.23**

© 2024 Davis SM et al. *JAMA Network Open.*

MH-PTSD	46	454	279	2106	0.76	1.06	0.55
**MH-Schizophrenia**	**16**	**484**	**38**	**2347**	**2.04**	**3.69**	**1.13**
MH-Social Phobia	16	484	84	2301	0.91	1.56	0.53
**MSK-Gout**	**39**	**461**	**351**	**2134**	**0.51**	**0.73**	**0.36**
MSK-Osteoarthritis	59	441	357	2028	0.76	1.02	0.57
MSK-Osteoporosis	14	486	74	2311	0.90	1.61	0.50
MSK-Other arthritis	125	375	542	1843	1.13	1.42	0.91
MSK-Other MSK problem	91	409	530	1855	0.78	1.00	0.61
MSK-Rheumatoid arthritis	41	459	190	2195	1.03	1.47	0.73
Neuro-ALS	2	498	1	2384	9.57	105.80	0.87
Neuro-Dementia	6	494	23	2362	1.25	3.08	0.51
Neuro-Epilepsy	18	482	54	2331	1.61	2.77	0.94
**Neuro-Loss of consciousness**	**65**	**435**	**231**	**2154**	**1.39**	**1.87**	**1.04**
Neuro-Memory loss/impairment	62	438	267	2118	1.12	1.51	0.84
**Neuro-Migraines**	**68**	**432**	**221**	**2164**	**1.54**	**2.06**	**1.15**
Neuro-MS	2	498	12	2373	0.79	3.56	0.18
**Neuro-Other**	**54**	**446**	**164**	**2221**	**1.64**	**2.27**	**1.19**
**Neuro-Other Headaches**	**87**	**413**	**256**	**2129**	**1.75**	**2.28**	**1.34**
Neuro-Parkinson's disease	10	490	27	2358	1.78	3.71	0.86
Neuro-Spinal Cord Injury	45	455	184	2201	1.18	1.66	0.84
Neuro-Traumatic Brain Injury	21	479	91	2294	1.11	1.79	0.68
Onc-Breast cancer	0	500	2	2383			
Onc-Colorectal cancer	8	492	40	2345	0.95	2.05	0.44
Onc-Lung cancer	9	491	29	2356	1.49	3.17	0.70
Onc-other cancer	34	466	173	2212	0.93	1.37	0.64
Onc-Prostate cancer	26	474	151	2234	0.81	1.24	0.53
Onc-Skin cancer	68	432	356	2029	0.90	1.19	0.68
Other disease/disorder	27	473	92	2293	1.42	2.21	0.92
Other-Benign Prostatic Hypertrophy	88	412	429	1956	0.97	1.25	0.76
**Pulm-Asthma**	**78**	**422**	**217**	**2168**	**1.85**	**2.44**	**1.40**
**Pulm-Chronic lung disease**	**86**	**414**	**289**	**2096**	**1.51**	**1.96**	**1.16**
**Pulm-Sleep apnea**	**174**	**326**	**664**	**1721**	**1.38**	**1.70**	**1.13**
**Renal-Acute Kidney Disease**	**12**	**488**	**27**	**2358**	**2.15**	**4.27**	**1.08**
Renal-Kidney Disease no dialysis	35	465	121	2264	1.41	2.08	0.95
**Renal-Kidney Disease with dialysis**	**12**	**488**	**29**	**2356**	**2.00**	**3.94**	**1.01**
Skin Condition	59	441	243	2142	1.18	1.60	0.87

The number of participants endorsing each of the 75 potential conditions from the Baseline Survey and calculated odds ratio (OR) with upper and lower 95% confidence intervals (U95%CI, L95CI). Bolded values are significantly different between SCA cases and controls at an alpha of 0.05

© 2024 Davis SM et al. *JAMA Network Open.*
