# Supplementary material for: Prevalence, Morbidity, and Mortality of Men With Sex Chromosome Aneuploidy in the Million Veteran Program Cohort
Source: JAMA Netw Open. 2024 Mar 29;7(3):e244113. doi: 10.1001/jamanetworkopen.2024.4113 (PMC10980972; doi:10.1001/jamanetworkopen.2024.4113)
Supplement: Supplement 2. — Data Sharing Statement [file jamanetwopen-e244113-s002.pdf]

Data Sharing Statement

Davis. Prevalence, Morbidity, and Mortality of Men with Sex Chromosome Aneuploidy in the Million Veteran Program Cohort. *JAMA Netw Open*. Published March 29, 2024. doi:10.1001/jamanetworkopen.2024.4113

Data
Data available: Yes
Data types: Deidentified participant data, Data (not involving human participants), Data dictionary
How to access data: Patient-level data are already accessible to all VA researchers with appropriate IRB approvals.
When available: With publication

Supporting Documents
Document types: None

Additional Information
Who can access the data: Patient-level data are currently accessible to all VA researchers with appropriate IRB approvals.
Types of analyses: Data is available for any type of analysis.
Mechanisms of data availability: Data will be made available after IRB approvals.
